# High sensitivity sanger sequencing detection of BRAF mutations in metastatic melanoma FFPE tissue specimens

**DOI:** 10.1038/s41598-021-88391-5

**Published:** 2021-04-27

**Authors:** Lauren Y. Cheng, Lauren E. Haydu, Ping Song, Jianyi Nie, Michael T. Tetzlaff, Lawrence N. Kwong, Jeffrey E. Gershenwald, Michael A. Davies, David Yu Zhang

**Affiliations:** 1 Department of Bioengineering, Rice University, 65000 Main St, Houston, TX 77030 USA; 2 Department of Surgical Oncology, The University of Texas M. D. Anderson Cancer Center, Houston, TX USA; 3 Department of Translational Molecular Pathology, The University of Texas M. D. Anderson Cancer Center, Houston, TX USA; 4 Department of Melanoma Medical Oncology, The University of Texas M. D. Anderson Cancer Center, Houston, TX USA; 5 Systems, Synthetic, and Physical Biology, Rice University, Houston, TX USA

**Keywords:** Oncogenes, Tumour biomarkers, Diagnostic markers

## Abstract

Mutations in the BRAF gene at or near the p. V600 locus are informative for therapy selection, but current methods for analyzing FFPE tissue DNA generally have a limit of detection of 5% variant allele frequency (VAF), or are limited to the single variant (V600E). These can result in false negatives for samples with low VAFs due to low tumor content or subclonal heterogeneity, or harbor non-V600 mutations. Here, we show that Sanger sequencing using the NuProbe VarTrace BRAF assay, based on the Blocker Displacement Amplification (BDA) technology, is capable of detecting BRAF V600 mutations down to 0.20% VAF from FFPE lymph node tissue samples. Comparison experiments on adjacent tissue sections using BDA Sanger, immunohistochemistry (IHC), digital droplet PCR (ddPCR), and NGS showed 100% concordance among all 4 methods for samples with BRAF mutations at ≥ 1% VAF, though ddPCR did not distinguish the V600K mutation from the V600E mutation. BDA Sanger, ddPCR, and NGS (with orthogonal confirmation) were also pairwise concordant for lower VAF mutations down to 0.26% VAF, but IHC produced a false negative. Thus, we have shown that Sanger sequencing can be effective for rapid detection and quantitation of multiple low VAF BRAF mutations from FFPE samples. BDA Sanger method also enabled detection and quantitation of less frequent, potentially actionable non-V600 mutations as demonstrated by synthetic samples.

## Introduction

Melanoma is the most aggressive of the common forms of skin cancer^1^. Treatment options for metastatic melanoma have increased drastically with development of immunotherapies and targeted therapies^2,3^. Immunotherapies (e.g. cytokines, PD-1 or PD-L1 inhibitors) can produce long-lasting responses but are only effective in a small fraction of patients^4–7^. Targeted therapies have been shown to be highly effective for people with specific oncogene mutations. In melanoma, the BRAF-V600E mutation, for example, is present in roughly 50% of patients^8,9^, and is indicative of positive clinical response to BRAF inhibitors^10–16^. To date, the detection of the BRAF-V600E mutation by a certified assay is required in order for stage IV metastatic melanoma patients to be prescribed targeted therapy treatment with dabrafenib and trametinib (FDA approval, 2014), vemurafenib and cobimetinib (FDA approval, 2015), or encorafenib and binimetinib (FDA approval, 2018).

A subset of melanomas harbor non-BRAF p. V600E mutations in codon 600 or its proximity (e.g. p. L597Q & K601E)^17–19^, and studies have shown efficacy for targeted therapies in metastatic melanoma patients with mutations that affect other residues in BRAF^20–23^, albeit at a lower response rate compared to V600E mutated cases^24^. These non-V600E mutations are not generally detectable by commercial quantitative PCR (qPCR) and digital PCR molecular diagnostic assays. For example, commercially available FDA cleared BRAF qPCR mutations tests, such as cobas 4800 BRAF V600 Mutation Test, THXID BRAF Kit, and therascreen BRAF V600E RGQ PCR Kit, cannot detect mutations below 1% and do not cover non-V600 mutations. The Droplet Digital PCR (ddPCR) V600 Screening Kit by Bio-Rad detects (but does not distinguish among) the p. V600E/K/R mutations, and likewise does not detect mutations in the 597 or 601 codons. Immunohistochemistry (IHC) method has high clinical sensitivity and specificity to V600E mutant, but is specific to just the BRAF p. V600E mutation.

**Figure 1:**
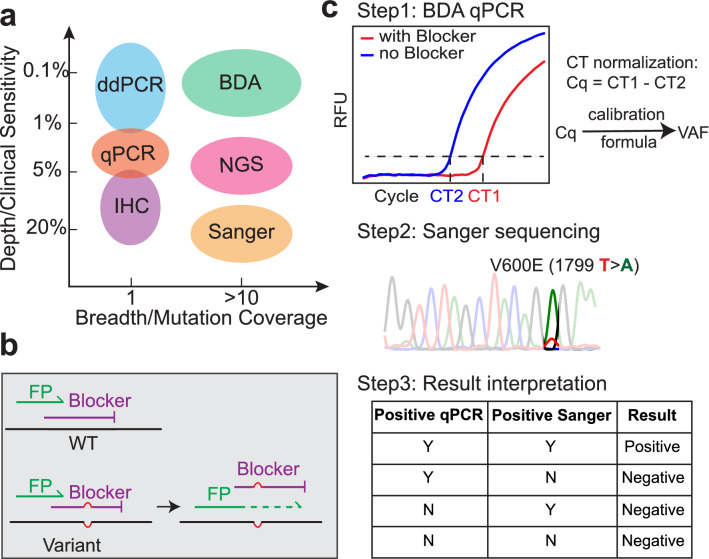
Methods for detection of BRAF mutations. (**a**) Comparison of depth and breadth of available BRAF mutation detection methods. (**b**) Mechanism for BDA variant enrichment^25^. The Blocker preferentially binds to and suppresses the PCR amplification of wildtype (WT) DNA sequences, resulting in selective amplification of BRAF mutations. (**c**) Workflow of BDA mutation detection assay. BDA is first performed in qPCR, and the amplicons are subsequently Sanger sequenced to identify the specific mutation.

Sequencing based methods are capable of detecting many different potential BRAF mutations. Sanger sequencing, considered the gold standard for clinical evaluation of BRAF hotspot mutations, can detect the full range of BRAF mutations but is limited in sensitivity to approximately 15%. High-throughput sequencing-by-synthesis (NGS) significantly improves the sensitivity, but still requires orthogonal confirmation for mutations below 5% variant allele frequency (VAF). Additionally, NGS has a turnaround time of roughly 1 week and is not economical for mutation analysis of a single hotspot. Thus, although multiple methods exist for BRAF mutation detection in DNA extracted from FFPE samples, they all under-serve the clinical need of rapid, sensitive, and comprehensive detection of actionable BRAF mutations (Fig. 1).

Here, we evaluate the effectiveness of high sensitivity Sanger sequencing for detection of low VAF BRAF mutations. We use the NuProbe VarTrace BRAF assay, in which Blocker Displacement Amplification (BDA) selectively amplifies BRAF DNA sequence variants^25–27^. Importantly, this approach can detect and quantitate more than 50 BRAF mutations in codons 596–601 (Supplementary Table S1) with 0.1% VAF limit of detection (LOD). We tested 35 formalin-fixed paraffin-embedded (FFPE) sentinel lymph node biopsy (SLNB) or completion lymph node dissection (CLND) samples from 30 patients using the BDA Sanger approach. Additional comparative analyses (ddPCR, NGS, and IHC) were performed on 12 samples and found high concordance for BRAF mutations down to 0.26% VAF. Based on our BDA Sanger findings, we further ruled out non-V600 mutations in these samples at ≥0.2% VAF; other methods did not provide this information so it was not possible to evaluate concordance. The short 1-day turnaround renders the BDA Sanger approach attractive for clinical decision-making, and the improved sensitivity further obviates the need for tissue macrodissection, saving pathologist time.

## Materials and methods

### Patients and study materials

35 specimens from 30 patients with cutaneous melanoma were retrospectively selected with the approval of the Institutional Review Board at the University of Texas MD Anderson Cancer Center. All methods were carried out in accordance with relevant guidelines and regulations, and informed consent was obtained from all subjects. Tissue specimens were collected via SLNB or CLND and prepared as FFPE blocks. 10 μm-thick serial sections were cut from FFPE blocks and collected on glass slides. A hematoxylin-and-eosin (H&E) stained slide was assessed by pathologists to mark tumor areas for each patient sample. For macrodissected samples, non-tumor cells were scrapped off using the H&E stained slides as reference. Samples for NGS, IHC, BDA Sanger and ddPCR assays were taken from the same biopsy or dissected tissue so adjacent slides were typically used for different analysis. BDA Sanger and ddPCR analysis aliquoted from the same DNA extract. Comparative analyses were performed on 12 FFPE DNA samples and BDA Sanger alone was performed on the remaining 23 samples with repaired FFPE DNA.

### DNA extraction from FFPE specimens

DNA extraction from FFPE specimens was performed using QIAamp DNA FFPE Tissue Kit (Qiagen, 56404) according to manufacturer’s protocol. DNA was eluted in approximately 15 μl of elution buffer. The yield and purity were measured by a NanoDrop spectrophotometer. DNA materials were stored at – 20 °C until ready for analysis.

### Repair of extracted FFPE DNA

Repair of extracted DNA was performed using NEBNext FFPE DNA Repair Mix (NEB, M6630S). Extracted DNA was brought to 53.5 μl with water, mixed with 6.5 μl of FFPE DNA Repair Buffer and 2 μl of NEBNext FFPE DNA repair mix. The mixture was incubated at 20 °C for 15 min, followed by DNA cleanup using 3× volume of AMPure XP beads (Beckman Coulter, A63881) and elute in 18 μl of water.

### Reference material preparation

50% BRAF V600E Reference Standard was purchased from Horizon Discovery (HD238) and was diluted with WT genomic DNA (Coriell, NA18537) to prepare reference samples of 10%, 5%, 3%, 1%, 0.5%, 0.2% and 0.1%. Synthetic gBlocks from IDT served as positive sample materials for the following non-V600E mutations: L597Q, L597R, L597S, V600K, V600R, and K601E. After serial dilutions using Carrier RNA (Qiagen, 1017647) solution as diluent to prevent adsorption to plasticware, synthetic gBlock concentration were estimated by qPCR, and then gBlocks were diluted to approximately 10,000 molecules/μl.

The Ct values of the synthetic gBlocks were compared to the Ct values of 50 ng per well gDNA assayed with the same primers, and the concentrations of the synthetic templates were estimated based on the Ct differences. Based on estimated molecular concentrations, 10% of reference samples were prepared by mixing quantitated gBlock and WT gDNA, and lower VAF reference samples were prepared by further diluted 10% reference samples with WT genomic DNA. To verify whether the mutation spike-ins were accurate, NGS libraries were constructed from 10% reference samples via PCR and ligation of PCR product using NEBNext Ultra II DNA Library Prep Kit for Illumina (E7645S) according to manufacturer’s protocol and sequenced on Illumina Miseq with greater than 10,000 depth for each sample. The sample VAF values were then corrected if NGS VAFs were off by more than 20%.

### VarTrace BDA qPCR assay

qPCR assay was performed according to user manual. Roughly 40 ng of FFPE-derived DNA in 6 μl were loaded as input into each reaction, and the BDA qPCR was performed on a Bio-Rad CFX96 instrument. BDA qPCR products were purified with ExoSAP-IT Express PCR Product Cleanup Reagents (Thermo Fisher, 75001) to digest residual primers and deactivate dNTPs. Cycle sequencing was performed using BigDye Terminator v3.1 Cycle Sequencing Kit (Thermo Fisher, 4337455). BigDye Terminator 3.1 Ready Reaction Mix, BigDye Terminator v1.1 & v3.1 5× Sequencing buffer, uni-directional sequencing primer, purified PCR product and nuclease-free water were mixed, and the total volume was 10 μl per reaction. Thermo cycling program started with 1 min polymerase activation at 96 °C, followed by 25 repeated cycles of 10 s at 96 °C for DNA denature, 5 s at 50 °C for annealing, and 2 min at 60 °C for extension, and the samples were held at 4 °C until ready to purify (96 °C:1 min–(96 °C:10 s–50 °C:5 s–60 °C:2 min) × 25–4 °C:hold). Ab1 files were read by A Plasmic Editor (ApE) software and mutation status was visually inspected by comparing Sanger traces with WT reference sequence at loci of interest.

For reference samples, BDA qPCR experiments used 30 ng of synthetic spike-in DNA with VAFs ranging from 100% down to 0.1%, and 30 ng of wild-type (WT) gDNA. Differences in Ct values from two reactions (termed Cq) were calculated and linear fit was implemented for Cq vs. log_10_(VAF) using Matlab Curve fitting application. The fitted linear coefficients established the formula (Cq = k × log_10_(VAF) + b, where k and b are fitted linear coefficients) for each mutation to calculate sample’s original VAF from qPCR Cq readout.

### BRAF V600 ddPCR quantitation

ddPCR BRAF V600 Screening Kit (Bio-Rad, 12001037) was used to quantitate hotspot BRAF V600 mutations following methods described in Ref.^25^. 20 μl of reaction mix containing 2× ddPCR Supermix, 20× BRAF V600 Screening Assay and roughly 40 ng of DNA sample were prepared and added to the DG8 cartridges (Bio-Rad, 1864008). With the addition of 70 μl Droplet Generation Oil for Probes (Bio-Rad, 1863005), QX200 Droplet Generator (Bio-Rad, 10031907) was used to produce droplet emulsion. Then PCR started with 10 min at 95 °C, followed by 40 cycles of 30 s at 94 °C for DNA denaturing and 1 min at 55 °C for annealing/extension, and ended with 10 min at 98 °C. The plate was then read by a QX200 Droplet Reader (Bio-Rad, 1864003) to collect droplet florescence data.

### NGS mutation analysis

Macrodissected, tumor-enriched FFPE specimens were submitted to MD Anderson Molecular Diagnostic Laboratory for NGS analysis using the CMS46 panel (Ion Torrent), CMS50 panel (Ion Torrent), or Solid Tumor Genomics Assay v1 (Illumina). Each NGS assay’s LOD for BRAF V600E was roughly 5% VAF. For samples with suspected BRAF mutations at below 5% VAF, an orthogonal NGS assay was used, and BRAF V600E mutation was qualitatively reported only if the orthogonal assay confirmed the mutation.

### Anti-BRAF V600E staining and imaging

BRAF V600E IHC staining with anti-BRAF V600E (clone VE1) was performed as previously described in Ref.^28^. In short, clone VE1 (Spring Bioscience) was diluted 50× and staining and imaging were done on an automated IHC staining instrument (Bond, Leica Biosystems).

### Image analysis

Aperio Cytoplasmic v2 algorithm was used to analyze anti-BRAF-stained images and quantitate expression of the variant BRAF protein. The “Cytoplasm: Percent Positive Cells” algorithm result was reported as percentage of cells carrying the BRAF V600E mutation.

### Ethics approval and consent to participate

Research was approved via Institutional Review Board (IRB) at the University of Texas MD Anderson Cancer Center (FWA00000363).

## Results

Metastatic tumor cells residing in lymph nodes are surrounded by a large number of leukocytes and stroma cells that do not contain genetic alterations, resulting in low tumor fraction. Furthermore, tumor genetic profile can evolve over time under various selective pressures, leading to tumor heterogeneity^27,29,30^. Consequently, actionable mutations can be at low VAFs, and assays with poor VAF limits of detection can exhibit clinical false negatives that deprive patients from optimal targeted therapies. To mitigate this problem, clinical pathology labs may enrich tumor content through labor- or capital-intensive macrodissection or laser microdissection. However, these approaches cannot overcome tumor subclonal heterogeneity developed by various tumor evolution mechanisms. Thus, an assay that detects a range of BRAF mutations with LOD below VAF of 1% will likely produce higher clinical sensitivity than current clinical practice. With the BDA Sanger approach, a range of BRAF mutations are detected in FFPE tissue-derived DNA with VAF down to 0.20%.

### Analytical and clinical validation of BRAF V600E mutation

We first validated the performance of the BDA BRAF assay on detecting and quantitating the most common BRAF V600E mutation using Horizon Discovery reference materials and synthetic DNA strands. We ran the BRAF assays on reference materials with VAF values ranging from 0.1% to 100%. Two reactions were performed for each sample, one for selective variant enrichment and the other for input quantitation. The normalized result from taking the difference of the two Ct values (Cq) is independent of DNA input and can be used to determine the mutation’s VAF. Higher VAFs in the reference material were reflected as earlier amplification and thus lower Cq values (Supplementary Fig. S1a).

Cq values and log VAFs exhibited linear correlation with an R^2^ greater than 0.99 for V600E. The equation derived from the linear fitting was used to quantitate unknown VAFs in the original sample (Supplementary Fig. S1b). Median Cq value of 0.1% reference sample was 10.2, and that of WT sample was 11.8, and value of Cq for WT was always at least 1.0 higher than that of the V600E sample at 0.1% VAF (Supplementary Fig. S1b). The Sanger sequencing trace of the qPCR amplicon product showed V600E at roughly 50% after BDA enrichment (Supplementary Fig. S1c), confirming the identity of the mutation.

Next, we applied the BDA BRAF assay and comparative analyses to 12 FFPE SLNB or CLND samples from seven metastatic melanoma patients (patient 1–7). For 5 of the patients, we prepared paired tumor-enriched/not enriched FFPE samples; paired tissues for other two patients were not available (Table 1). Results showed that 7 FFPE specimens from 4 patients (patient 1–4) had V600E mutations with VAFs ranging from 0.26% to 38.30%. The two specimens from patient 6 were identified as V600-WT. The remaining two specimens from patient 5 had two separate G > A variants (Supplementary Fig. S2d) that are characteristic of deamination damage associated with FFPE treatment and storage. Because the incidence of two G > A mutations appearing simultaneously in the same sample in such close proximity is low, we do not believe these are real mutations. More generally, identification of multiple G > A or C > T mutations in the same sample in close proximity are likely to be hallmarks of unrepaired FFPE damage.

**Table 1:**
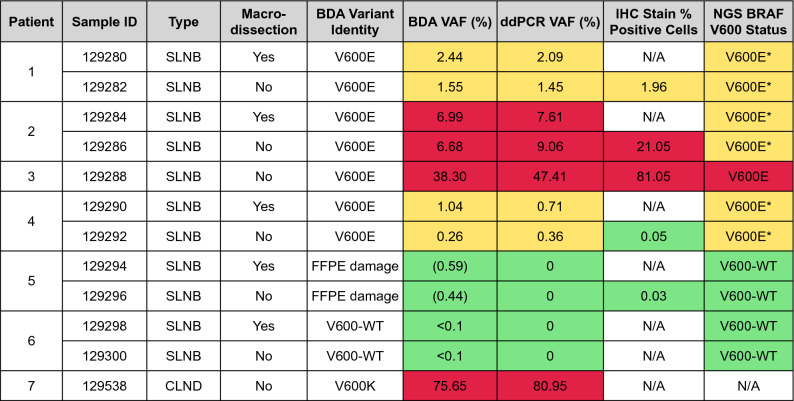
Clinical sample summary of comparative analytical results. DNA was extracted from FFPE SLNB or CLND specimens from non-acral cutaneous melanoma patients.

Samples were derived from 7 patients. Macrodissection was performed to enrich tumor fraction except for samples 129288 and 129538 that were tumor only. Samples results are shown for BDA Sanger sequencing assay, ddPCR assay, IHC using anti-BRAF V600E antibody staining, and NGS. DNA input for BDA Sanger and ddPCR assays were 40ng each (roughly 1/50th the DNA from 1 FFPE slide); other methods used a full slide. Red cells code for VAF 5%, yellow cells code ≥ for 0.1% ≤ VAF < 5%, green cells code for wild type. BDA Sanger identified and quantitated FFPE damage at 0.59% VAF and 0.44% VAF for the 129294 and 129296 samples, but reported 0% VAF for BRAF actionable mutations. “*” indicated that the NGS status was confirmed with orthogonal NGS assay.

**Figure 2:**
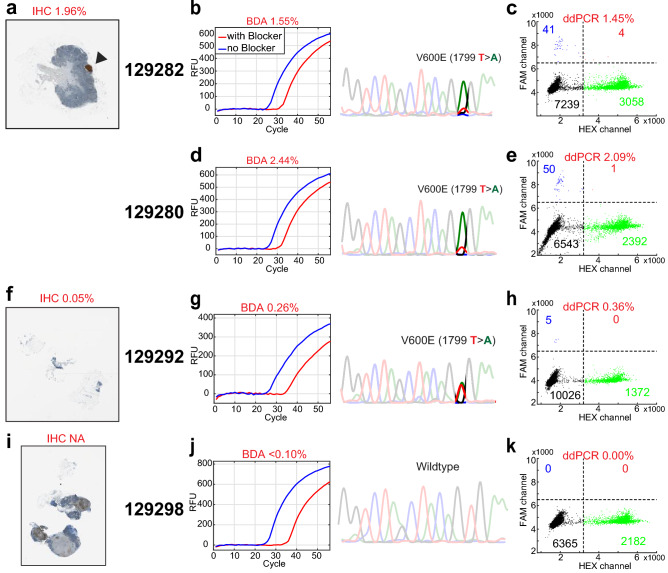
Comparative analytical results for select FFPE clinical samples. (**a**) IHC anti-BRAF V600E stained tissue from patient 1. Stained tumor exhibited brown color as pointed by the arrowhead. (**b**,**c**) Results for non-macrodissected patient 1 sample 129282, including BDA qPCR curves, Sanger sequencing trace(b) and ddPCR scatter plot(c). (**d**,**e**) Results for macrodissected patient 1 sample 129280. (**f**–**h**) IHC, BDA Sanger, and ddPCR results for patient 4 sample 129292. (**i**–**k**) IHC, BDA Sanger, and ddPCR results for patient 6 sample 129298. Red qPCR curve showed amplification of BDA reaction with blocker for variant enrichment, and blue curve showed amplification of control reaction that did not contain blocker. The position of variant peak is highlighted in Sanger trace. In ddPCR scatter plots, the lower left quadrant showed empty droplets. The lower right quadrant showed droplets containing WT molecules. The upper left quadrant displayed droplets containing variant molecules. The upper right quadrant exhibited droplets containing both WT and variant molecules.

**Figure 3:**
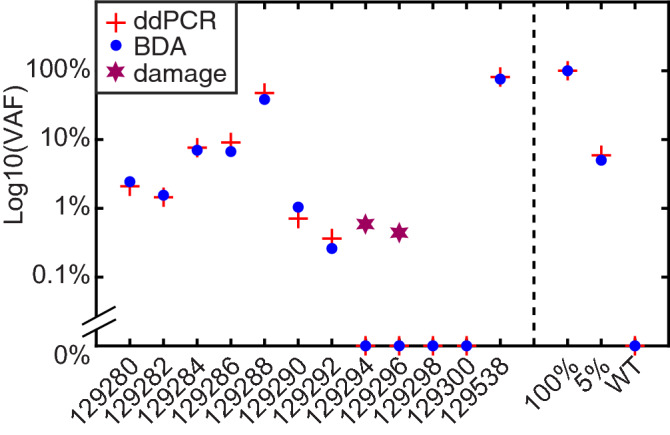
BDA and ddPCR mutation quantitation comparison. “+” symbols refer to ddPCR determined VAF values and blue dots refer to BDA determined VAFs for actionable BRAF mutations. Purple stars refer to FFPE damage profiled by the BDA Sanger assay. The right three samples are reference control samples with BRAF V600E at 100%, 5%, and 0% VAF.

The comparative analyses include ddPCR BRAF V600 screening kit from BioRad, anti-BRAF V600E IHC staining and NGS on matched tumor samples from the same individuals (Table 1). For a low tumor fraction sample of patient 1 (Fig. 2a), macrodissection was able to increase tumor fraction as seen in both BDA quantification results (Fig. 2b,d) and ddPCR results (Fig. 2c,e). The lowest VAF detected by BDA assay was 0.26% in sample 129292, which had VAF of 0.36% assayed by ddPCR. Sanger trace of the same sample showed that the ultra-low level variant was enriched by BDA assay to approximately 50% (Fig. 2f–h), allowing Sanger to visualize low level mutations. For all V600E-positive specimens identified by BDA assay, ddPCR results were all positive and showed high quantitative concordance with BDA assay even at VAF levels lower than 1% (Table 1, Fig. 3). ddPCR did not detect any variant molecule in samples with no BRAF V600 mutation according to BDA assay. Nevertheless, suspected FFPE damages at nearby loci were not detected by ddPCR as it could not detect mutations outside codon 600 (Supplementary Fig. S2d).

For samples with mutation VAFs greater than 1%, BDA Sanger, ddPCR, and IHC all had high quantitative concordance. For example, sample 129288 had VAF of 38.30% identified by BDA, which is consistent with a heterozygous mutation in 81.05% of cells identified through IHC (Table 1). It is also worth noting that IHC could not be used to find V600E positive cells for patient 6, because it was pigmented and melanin produced similar brown color that would be mistaken as positively stained in DAB (3,3′-diaminobenzidine) detection system (Fig. 2i). All three molecular diagnostic approaches (BDA Sanger, ddPCR, and NGS) do not have this limitation (Fig. 2j,k).

The BRAF mutation status for these samples were qualitatively reported by MD Anderson Molecular Diagnostic Laboratory, which applied one of three separate targeted NGS panels on the Ion Torrent or Illumina NGS platforms^32^. These panels typically have a limit of detection of about 5% VAF, but given the importance of the BRAF V600E mutation, samples with sub-threshold V600E VAFs were re-analyzed by a second NGS panel using a different sequencing platform. Because Illumina and Ion Torrent are based on different detection principles (optical fluorescence vs. pH) and have different error profiles, it was unlikely that the same false positive variants would appear in both platforms. Thus, if a sample analyzed by both NGS platforms contained reads supporting the V600E mutation, then the mutation was called regardless of implied VAF.

To address the issue with the observed FFPE damage, 23 samples of FFPE DNA were repaired using the NEBNext FFPE DNA Repair Mix prior to the BDA Sanger assay (Supplementary Table S3). Out of the 23 repaired FFPE samples, eight samples were identified as BRAF V600E mutated and the lowest level of detected V600E mutation is 0.20% (Supplementary Fig. S3). Nine samples were identified as BRAF V600-WT (Supplementary Fig. S5), and out of which the characteristic base change from FFPE artifact is only observed in one sample, with 0.43% VAF of G > A substitution in sample 129513. In other wild-type samples, although their Sanger traces exhibited substitutions, the VAF level determined from BDA qPCR quantitation are below the 0.1% detection limit and thus would not be reported as positive. The substitutions in wild-type Sanger traces are likely caused by polymerase misincorporation or low-level damage not repaired by the cocktail of repair enzymes.

### Analytical and clinical characterization of BRAF non-V600E mutations

**Figure 4:**
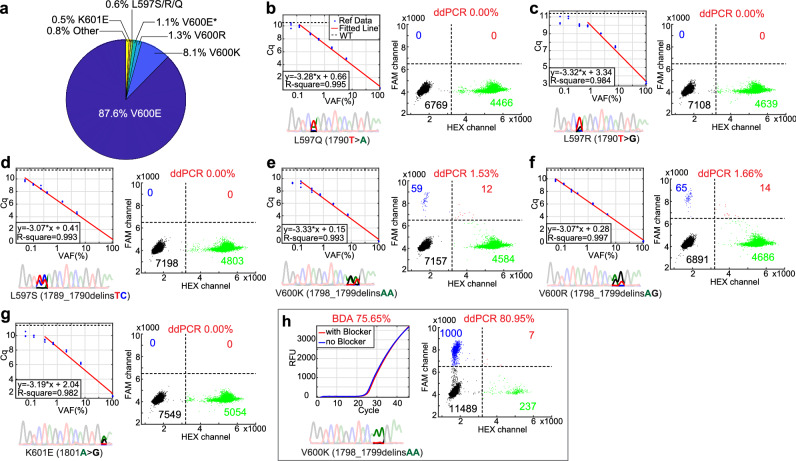
Analytical and clinical testing of non-V600E BRAF mutations. (**a**) BRAF mutation distribution in melanoma. Melanoma-associated BRAF mutation data was retrieved from the COSMIC database^31^ and mutations with total counts greater than 10 were collected and plotted. Non-V600E mutations make up more than 10%. (**b**–**g**) Characterization of BDA Sanger performance on non-V600E BRAF mutations. Figures show qPCR Cq calibration, Sanger sequencing of low abundance reference material and ddPCR results for L597R, L597Q, L597S, V600K, V600R and K601E mutations. Triplicate qPCR data points were plotted as individual blue dots. The Cq values and logarithmic VAF values of reference samples were used to generate fitted red line. Cq values for WT samples were shown as dashed black line. Sanger traces are shown for 0.5% VAF spike-in reference samples. ddPCR plots are shown for 2% VAF spike-in samples; ddPCR was unable to make mutation calls for non-V600 mutations, as expected. (**h**) Results for non-macrodissected patient 7 sample 129538. BDA Sanger reported a V600K mutation whereas ddPCR did not identify the specific mutation.

Based on the COSMIC database, more than 10% of BRAF mutations in melanoma are non-V600E mutations (Fig. 4a) and some (i.e., V600R, L597Q/R/S, K601E) have been recently reported to be associated with efficacy of BRAF inhibitor therapy^24^. The BDA Sanger assay is in principle capable of detecting these mutations, and the lack of corresponding mutation peaks indicates that the samples tested are likely negative for all other BRAF mutations in this region.

To confirm the presumed result, we performed analytical validation of the BDA Sanger assay using spike-in reference samples with the L597R, L597Q, L597S, V600K, V600R, and K601E mutations (Fig. 4b–g). The limit of detection for all tested mutations were no worse than 0.5% VAF, and for most mutations 0.1% VAF would be confidently called based on qPCR Cq value alone (Fig. 4b–g). The quantitation formula generated from linear fitting varied for different mutations, suggesting that the variant enrichment performance was mutation-specific.

The BRAF V600K mutation is covered by the ddPCR BRAF V600 screening kit; consequently, the BDA Sanger and ddPCR assays were concordant for sample 129538 from patient 7 (Fig. 4h). However, because ddPCR is limited to a single fluorescence color channel for reporting all mutations in the kit, the V600K mutation in patient 7 could not be distinguished from the V600E mutations in patients 1 through 4. Additional five V600K samples and one V600R mutations were identified from the set of 23 repaired FFPE DNA samples, with VAFs range from 8.92 to 94.12% (Supplementary Fig. S4).

## Discussion

In this work, we demonstrated that the BDA Sanger approach can detect and quantify BRAF mutations in codons 596–601 with sensitivity down to 0.20% VAF in clinical FFPE samples. Although the samples we tested did not contain them, the method is also capable of detecting less frequent, potentially actionable mutations such as L597R, L597Q, L597S, V600K, V600R, and K601E. Future studies with larger cohorts will be important for studying the incidence of non-V600 BRAF mutations in melanoma patients, and the impact of such mutations on the outcomes of patients with BRAF inhibitor or other targeted therapies.

DNA deamination damage in FFPE tissue samples is a well-documented phenomenon^33^, and generally the amount of damage correlates with the age of the sample. Thus, we expect that the VAF limit of detection may be bottlenecked by FFPE damage for older samples. Repaired FFPE DNA showed reduced cases DNA deamination damage, and, therefore, could be employed as a standard procedure into FFPE DNA analysis workflow. Fresh/frozen tumor tissue samples would not have this limitation, and thus could potentially allow even better VAF limits of detection. However, fresh/frozen tumor tissue are not typically available as part of the standard clinical workflow, particularly as part of retrospective cohorts.

The rapid turnaround of the qPCR and Sanger workflows allow same day results reporting, which is not currently achievable by NGS. Furthermore, the high sensitivity of the assay simplifies tumor tissue analysis by eliminating the need to perform macro-/micro-dissection to enrich tumor fraction. For the three BRAF mutation positive patient samples in which we had both non-macrodissected and macrodissected tissues, the VAF for the latter was observed to be 1.57, 1.05 and 4.00 times as high as the former. The variation in enrichment is believed to be primarily due to differential tissue composition. Since macro-/micro-dissection is time-consuming, elimination of this step without loss of clinical sensitivity could reduce the total turnaround time.

BRAF mutations are also frequently observed in many other cancer types, including thyroid gland papillary carcinoma, colon adenocarcinoma, lung adenocarcinoma, breast invasive ductal carcinoma, and bladder urothelial carcinoma^34^. The high sensitivity of the BDA Sanger assay, combined with the short amplicon lengths, render it potentially effective for guiding therapy from multiple biospecimen types. For patients where tumor biopsy tissue samples are unavailable, “liquid” biopsy analysis based on cell-free DNA in peripheral blood plasma, saliva, or urine may serve as an effective substitute^35,36^.

## Supplementary Information

Supplementary Information.

## Data Availability

The datasets during and/or analyzed during the current study available from the corresponding author on reasonable request.
