# Supplementary material for: High sensitivity sanger sequencing detection of BRAF mutations in metastatic melanoma FFPE tissue specimens
Source: Sci Rep. 2021 Apr 27;11:9043. doi: 10.1038/s41598-021-88391-5 (PMC8079675; doi:10.1038/s41598-021-88391-5)
Supplement: Supplementary file 1 — Supplementary Information. [file 41598_2021_88391_MOESM1_ESM.pdf]

High Sensitivity Sanger Sequencing Detection of BRAF Mutations in Metastatic Melanoma FFPE Tissue Specimens

Lauren Y. Cheng,[1] Lauren E. Haydu,[2] Ping Song,[1] Jianyi Nie,[1] Michael T. Tetzlaff,[3]
Lawrence N. Kwong,[3] Jeffrey E. Gershenwald,[2] Michael A. Davies,[4] and David Yu Zhang[1,5]

[1]*Department of Bioengineering, Rice University, Houston, TX*
[2]*Department of Surgical Oncology, The University of Texas M. D. Anderson Cancer Center, Houston, TX*
[3]*Department of Translational Molecular Pathology, The University of Texas M. D. Anderson Cancer Center, Houston, TX*
[4]*Department of Melanoma Medical Oncology, The University of Texas M. D. Anderson Cancer Center, Houston, TX*
[5]*Systems, Synthetic, and Physical Biology, Rice University, Houston, TX*

S1. BRAF mutations covered by VarTrace^{TM} BRAF assay 1
S2. Characterization of BRAF V600E mutation using BDA Sanger BRAF assay 2
S3. IHC, BDA Sanger and ddPCR results of FFPE samples from melanoma patients 3
S4. BDA Sanger results of repaired FFPE samples from melanoma patients 5

Section S1: BRAF mutations covered by VarTrace^{TM} BRAF Assay

The BDA Sanger BRAF Assay is designed to detect and quantitate mutations in codons 596-60, covering 57 BRAF mutations reported on COSMIC database (Table. S1).

	CDS Position	CDS Mutation	AA Mutation	Legacy Mutation ID
1	596	c.1786G>C	p.G596R	COSM469
2	596	c.1787G>A	p.G596D	COSM26506
3	596	c.1786G>T	p.G596C	COSM6936824
4	596	c.1787del	p.G596Vfs*2	COSM36922
5	597	c.1790T>G	p.L597R	COSM471
6	597	c.1790T>A	p.L597Q	COSM1125
7	597	c.1789_1790delinsTC	p.L597S	COSM1126
8	597	c.1789C>G	p.L597V	COSM470
9	597	c.1791A>G	p.L597=	COSM1124
10	597	c.1789C>T	p.L597=	COSM133632
11	597	c.1790T>C	p.L597P	COSM1448593
12	598	c.1793C>T	p.A598V	COSM21549
13	598	c.1792G>A	p.A598T	COSM28505
14	598	c.1794T>A	p.A598=	COSM1448592
15	599	c.1794_1796dup	p.T599dup	COSM30730
16	599	c.1795_1797dup	p.T599dup	COSM144982
17	599	c.1794_1795insGTT	p.A598_T599insV	COSM26625
18	599	c.1796C>T	p.T599I	COSM472
19	599	c.1797A>G	p.T599=	COSM1448591
20	599	c.1796_1799delinsTAAA	p.T599_V600delinsIK	COSM1735763
21	599	c.1796C>G	p.T599R	COSM4172020
22	599	c.1797A>C	p.T599=	COSM3634274
23	599	c.1797A>T	p.T599=	COSM24963
24	599	c.1797_1798ins?	p.T599_V600insTT	COSM26459
25	599	c.1795_1796insTAA	p.A598_T599insI	COSM5881461
26	599	c.1795_1796insAAAAAATAGGTGATTTTGGTCTAGCTA	p.A598_T599insKKIGDFGLA	COSM4172575
27	599	c.1794_1796del	p.T599del	COSM1169497
28	600	c.1799T>A	p.V600E	COSM476
29	600	c.1798_1799delinsAA	p.V600K	COSM473
30	600	c.1798_1799delinsAG	p.V600R	COSM474
31	600	c.1799_1800delinsAA	p.V600E	COSM475
32	600	c.1799_1801del	p.V600_K601delinsE	COSM1133
33	600	c.1798G>A	p.V600M	COSM1130
34	600	c.1799_1800delinsAT	p.V600D	COSM477
35	600	c.1799T>C	p.V600A	COSM18443
36	600	c.1799T>G	p.V600G	COSM6137
37	600	c.1798G>C	p.V600L	COSM219798
38	600	c.1800G>T	p.V600=	COSM1578949
39	600	c.1798G>T	p.V600L	COSM33808
40	600	c.1797delinsTACTACG	p.T599_V600insTT	COSM1128
41	600	c.1797_1803delinsTGAGAAT	p.V600_K601delinsEN	COSM4389226
42	600	c.1800G>A	p.V600=	COSM249890
43	600	c.1798_1799delinsCA	p.V600Q	COSM249889
44	600	c.1798_1799delinsCG	p.V600R	COSM1583011
45	600	c.1798_1799inv	p.V600T	COSM5985086
46	600	c.1797_1799delinsGAG	p.V600R	COSM1127
47	600	c.1799_1800delinsAC	p.V600D	COSM308550
48	600	c.1798_1799ins6	p.T599_V600ins2	COSM6005496
49	600	c.1798_1799insAGGCTACAG	p.T599_V600insEAT	COSM4166148
50	600	c.1798_1799insAGACTACAG	p.T599_V600insETT	COSM4172018
51	600	c.1798_1800dup	p.V600dup	COSM7351000
52	600	c.1799_1800del	p.V600Efs*11	COSM1168053
53	601	c.1801A>G	p.K601E	COSM478
54	601	c.1801_1803del	p.K601del	COSM30594
55	601	c.1802A>C	p.K601T	COSM3878760
56	601	c.1802A>T	p.K601I	COSM26491
57	601	c.1801_1802inv	p.K601L	COSM6853796

Table. S1: List of BRAF mutations covered by BDA Sanger BRAF Assay.

Section S2: Characterization of BRAF V600E mutation using BDA Sanger BRAF Assay

We validated the performance of BDA Sanger BRAF assay on detecting and quantitating the most common BRAF V600E mutation using Horizon Discovery reference materials and synthetic DNA strands. qPCR and Sanger results showed final VAFs that were dramatically higher than the sample variant VAFs (Fig. S1), with 0.1% VAF enriched to over 50% VAF.

Fig.S1a shows triplicate PCR amplification curves of reference samples with VAF ranging from 0.1% to 100%, and wild-type samples. Fig.S1b shows linear fitting of Cq values and log VAF values, providing VAF quantitation formula for BRAF V600E mutation. Fig.S1c shows the Sanger trace of reference sample with 0.1% VAF after BDA PCR enrichment.

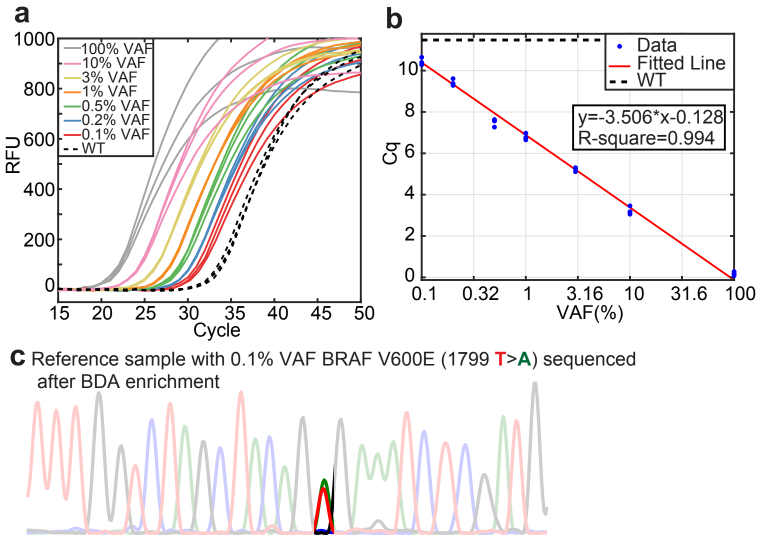

Fig. S1: Characterization of V600E mutation using VarTrace^{TM} qPCR and Sanger assay. **(a)** qPCR amplification plot of reference materials with known allele frequency values. **(b)** qPCR amplification calibration curve of V600E mutation. Triplicate qPCR data points were plotted as individual blue dots. The Cq values and log VAF values of reference samples were used to generate fitted red line. Cq value for wild-type sample was shown as black dashed line. **(c)** Sanger trace of 0.1% VAF reference sample.

Section S3: IHC, BDA Sanger and ddPCR results of FFPE samples from melanoma patients

We applied the BDA BRAF assay to 12 clinical samples from seven melanoma patients. Tissue specimens were collected via sentinel lymph node biopsy (SLNB) or completion lymph node dissection (CLND) and prepared as formalin-fixed, paraffin-embedded (FFPE) blocks. IHC, BDA Sanger and ddPCR results were qualitatively concordant. Fig.S2 shows IHC, BDA Sanger and ddPCR results of paired tumor-enriched/not enriched samples from patients 2, 4, 5, 6 and results of tumor-only sample from patient 3. The original sample VAF ranged from <0.1% to 38.40% and were all quantitated by BDA assay. Sanger traces showed that sample VAF were enriched by 100-fold after BDA qPCR reactions.

Patient	Age	Gender	Cancer Stage	Sample Year	Sample ID	Macro-dissection
1	51	F	IIIA	2014	129280	Yes
					129282	No
2	60	M	IIIC	2016	129284	Yes
					129286	No
3	75	M	IIIC	2016	129288	No
4	43	M	IIIA	2016	129290	Yes
					129292	No
5	65	M	IIA	2013	129294	Yes
					129296	No
6	51	F	IIIB	2013	129298	Yes
					129300	No
7	-	-	-	2013	129538	No

Table. S2: Patient information including age, gender, melanoma stage, tissue collection year and tumor-enrichment status.

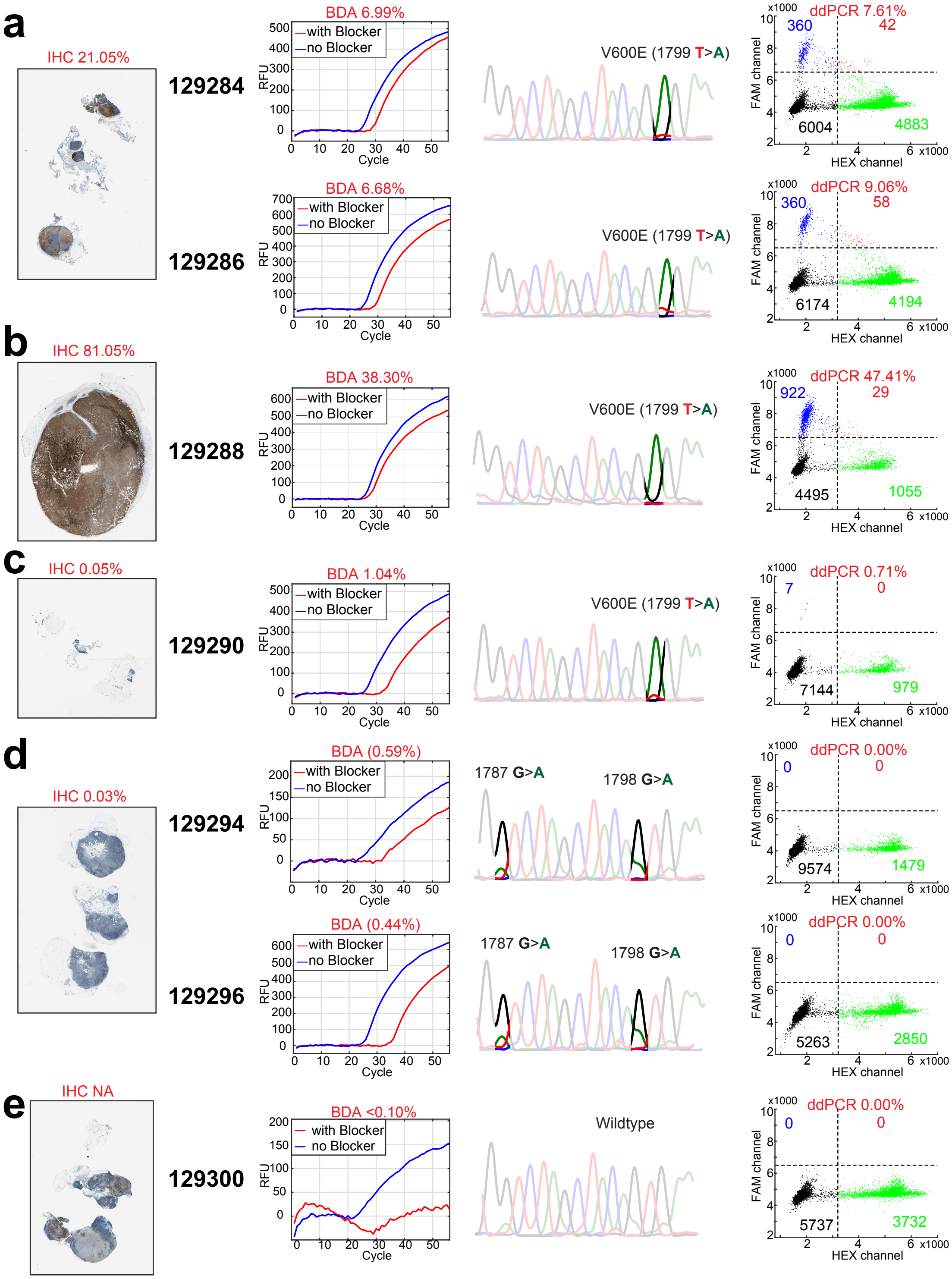

Fig. S2: IHC, BDA Sanger and ddPCR results of FFPE samples from melanoma patients. **(a)** Patient 2. Upper panel shows sample 129284 (tumor-enriched)and lower panel shows sample 129286 (not tumor-enriched). **(b)** Patient 3 sample 129288. This sample is tumor-only. **(c)** Patient 4. Upper panel shows sample 129290 (tumor-enriched) and lower panel shows sample 129292 (not tumor-enriched). **(d)** Patient 5. Upper panel shows sample 129294 (tumor-enriched) and lower panel shows sample 129296 (not tumor-enriched). **(e)** Patient 6 sample 129300 (not tumor-enriched). Samples of patient 6 were melanin-pigmented, the brown color in IHC image did not imply true positive staining. Brown spots (except for patient 6) in IHC images indicated areas with BRAF V600E mutation. Ct values of separate BDA qPCR reactions ("with Blocker" and "without Blocker") could be used to infer VAF quantity. In ddPCR scatter plots, the lower left quadrant showed empty droplets. The lower right quadrant showed droplet containing wild-type molecules. The upper left quadrant displayed droplets containing variant molecules. The upper right quadrant exhibited droplets containing both wild-type and variant molecules.

Section S4: BDA Sanger results of repaired FFPE samples from melanoma patients

We applied the BDA BRAF assay to 23 clinical samples from 23 melanoma patients. Tissue specimens were collected via sentinel lymph node biopsy (SLNB) and prepared as formalin-fixed, paraffin-embedded (FFPE) blocks. The FFPE tissue specimens were not macrodissected to increase tumor content. To reduce artifact from FFPE processing and aging, the FFPE samples were repaired prior to the assay. The mutation status and VAF quantitation results are exhibited in Table. S3. Fig.S3 shows BDA Sanger results of 8 samples with BRAF V600E mutation. Fig.S4 shows BDA Sanger results of 6 samples with BRAF non-V600E mutation. Fig.S5 shows BDA Sanger results of 9 samples with BRAF V600-WT status.

Patient	Sample ID	Type	Macro-dissection	FFPE Repair	BDA Variant Identity	BDA VAF (%)
8	129513	SLNB	No	Yes	FFPE damage	(0.43)
9	129514	SLNB	No	Yes	V600-WT	<0.1
10	129515	SLNB	No	Yes	V600E	4.56
11	129516	SLNB	No	Yes	V600E	7.30
12	129518	SLNB	No	Yes	V600E	6.72
13	129519	SLNB	No	Yes	V600-WT	<0.1
14	129520	SLNB	No	Yes	V600E	13.63
15	129522	SLNB	No	Yes	V600E	0.35
16	129523	SLNB	No	Yes	V600-WT	<0.1
17	129525	SLNB	No	Yes	V600-WT	<0.1
18	129529	SLNB	No	Yes	V600-WT	<0.1
19	129531	SLNB	No	Yes	V600-WT	<0.1
20	129532	SLNB	No	Yes	V600-WT	<0.1
21	129533	SLNB	No	Yes	V600E	0.20
22	129537	SLNB	No	Yes	V600-WT	<0.1
23	130024	SLNB	No	Yes	V600E	6.28
24	130025	SLNB	No	Yes	V600E	13.91
25	130026	SLNB	No	Yes	V600K	94.12
26	130037	SLNB	No	Yes	V600K	40.51
27	130070	SLNB	No	Yes	V600K	31.28
28	130075	SLNB	No	Yes	V600K	8.92
29	130076	SLNB	No	Yes	V600K	26.84
30	130080	SLNB	No	Yes	V600R	18.09

Table. S3: Clinical sample BDA Sanger results summary for repaired FFPE specimens. DNA was extracted from FFPE SLNB specimens from non-acral cutaneous melanoma patients and repaired using NEBNext FFPE Repair Mix (M6630S) prior to the BDA Sanger assay. Red cells code for VAF $\geqslant$ 5%, yellow cells code for 0.1% $\leqslant$ VAF $<$ 5%, green cells code for wild type. BDA Sanger identified and quantitated FFPE damage at 0.43% VAF for sample 129513, but reported 0% VAF for BRAF actionable mutations.

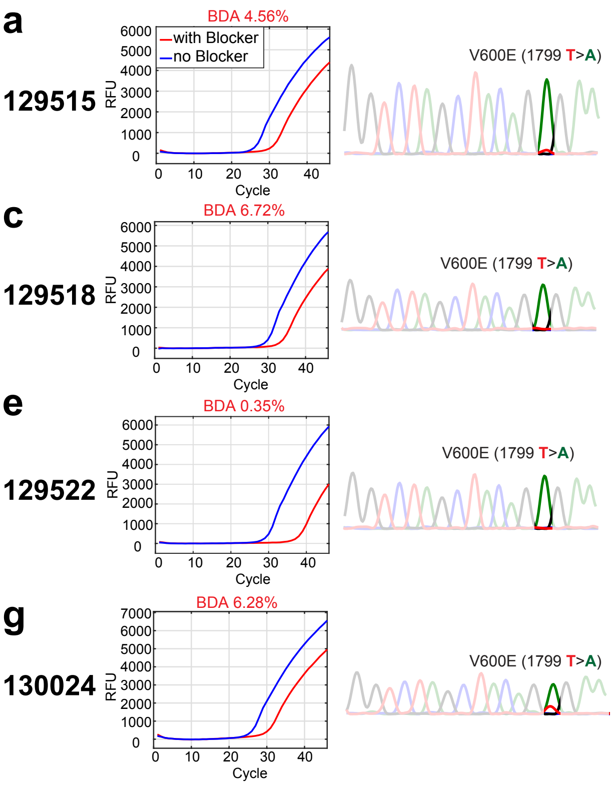

Fig. S3: BDA Sanger results of FFPE samples with BRAF V600E mutation. **(a)** Patient 10 sample 129515. **(b)** Patient 11 sample 129516. **(c)** Patient 12 sample 129518. **(d)** Patient 14 sample 129520. **(e)** Patient 15 sample 129522. **(f)** Patient 21 sample 129533. **(g)** Patient 23 sample 130024. **(h)** Patient 24 sample 130025. BDA inferred VAF values were determined from Cts of separate BDA qPCR reactions ("with Blocker" and "without Blocker").

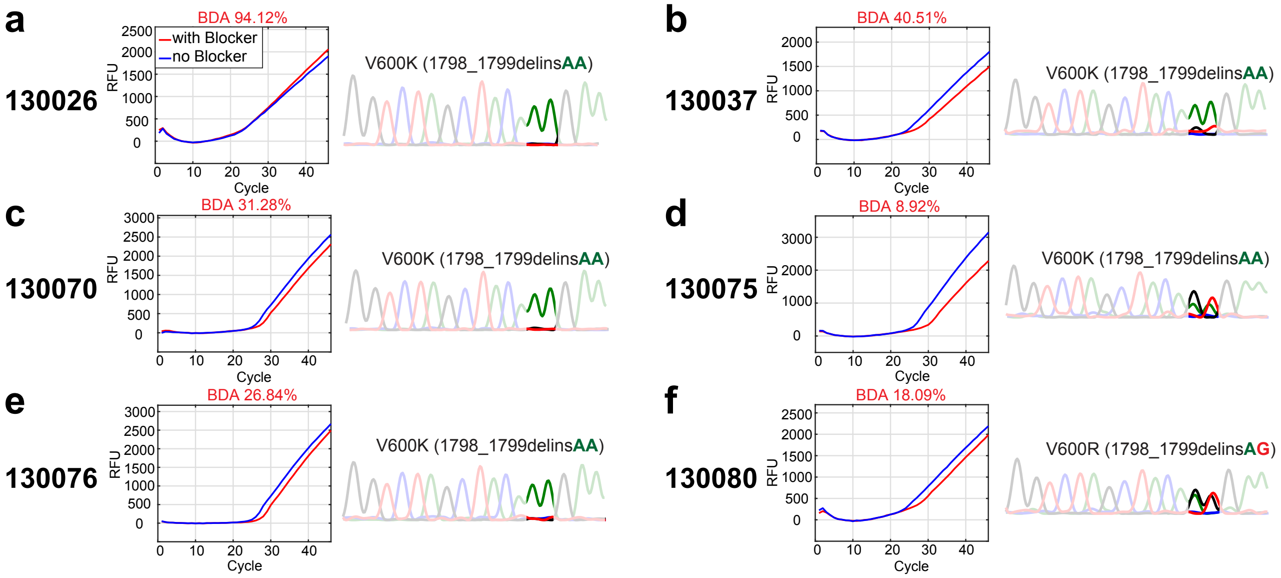

Fig. S4: BDA Sanger results of FFPE samples with BRAF non-V600E mutations. **(a)** Patient 25 sample 130026. **(b)** Patient 26 sample 130037. **(c)** Patient 27 sample 130070. **(d)** Patient 28 sample 130075. **(e)** Patient 29 sample 130076. **(f)** Patient 30 sample 130080. BDA inferred VAF values were determined from Cts of separate BDA qPCR reactions ("with Blocker" and "without Blocker").

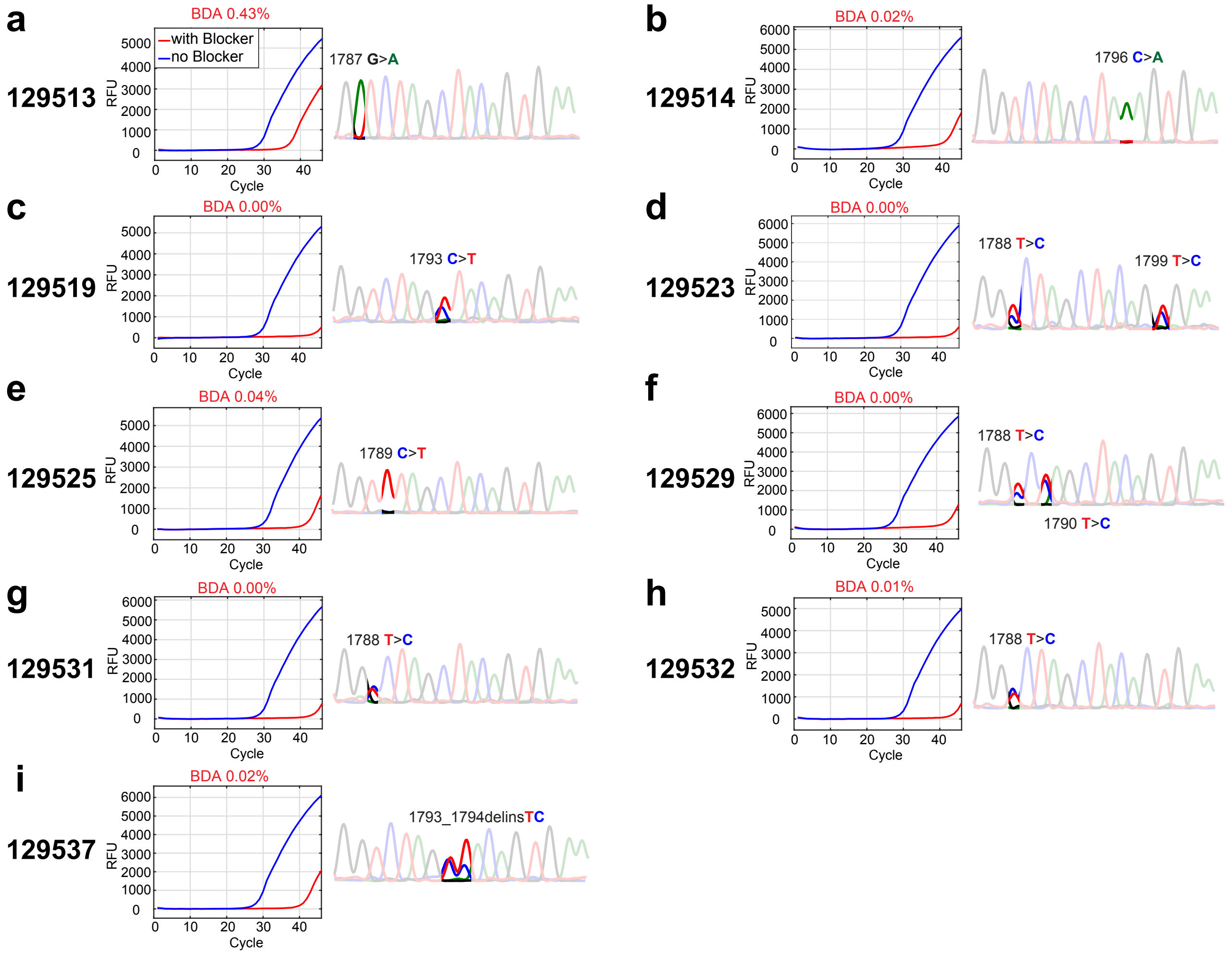

Fig. S5: BDA Sanger results of FFPE samples with BRAF V600-WT status. **(a)** Patient 8 sample 129513. The 0.43% G¿A base change was considered FFPE DNA artifact. **(b)** Patient 9 sample 129514. **(c)** Patient 13 sample 129519. **(d)** Patient 16 sample 129523. **(e)** Patient 17 sample 129525. **(f)** Patient 18 sample 129529. **(g)** Patient 19 sample 129531. **(h)** Patient 20 sample 129532. **(i)** Patient 22 sample 129537. BDA inferred VAF values were determined from Cts of separate BDA qPCR reactions ("with Blocker" and "without Blocker"). VAF below the limit of detection (0.1%) was considered wild-type.
